# Optimizing Implant Placement in Cases of Limited Interocclusal Space: Strategies and Challenges

**DOI:** 10.7759/cureus.60886

**Published:** 2024-05-23

**Authors:** Surekha A Dubey, Jahnavi P Gorripati, Sharayu Nimonkar, Madhu Priya

**Affiliations:** 1 Department of Prosthodontics, Sharad Pawar Dental College, Datta Meghe Institute of Medical Sciences, Wardha, IND

**Keywords:** screw retained prosthesis, fixed partial denture, vertical space, interocclusal space, implant-supported dental prosthesis

## Abstract

Effective treatment planning is crucial for implant-supported dental prostheses' success, requiring a thorough assessment of various factors, including bone quality, quantity, and available space. Evaluating space availability, encompassing height, width, and angulation, is imperative to ensure optimal implant positioning devoid of anatomical limitations. Adequate vertical space is essential for accommodating the implant-supported restoration while preserving proper occlusal function and esthetics. However, not all cases adhere to ideal standards, especially those featuring limited interocclusal space, as seen in scenarios of long-standing edentulous areas lacking prior prosthetic rehabilitation. Ideally, the interocclusal space should measure between 8-12 mm vertically. This case report details the management of reduced interocclusal space through the strategic placement of deeply positioned implants and the incorporation of a screw-retained fixed partial denture, effectively addressing the challenges associated with limited space.

## Introduction

Impressively, dental implants boast a 10-year survival rate of 95% in clinical practice, making them integral to modern dentistry [1,2]. Adequate interocclusal space is crucial for successful implant placement, as emphasized by Misch CE [3], who recommends a minimum vertical distance of 8-12 mm. However, restoring unrestored edentulous spaces resulting from posterior tooth loss presents challenges, leading to unpredictable movement of adjacent teeth and complicating traditional prosthodontic approaches with standard dental implants.

When faced with reduced interocclusal space during dental implant procedures, implantologists employ sophisticated strategies to optimize placement. These include surgical restoration of edentulous space through posterior maxillary segmental osteotomy [4,5], reduction of over-erupted opposing teeth [6], orthodontic treatment to retract extruded teeth [6,7], and utilization of an integrated crown-supported, screw-retained cast abutment with an implant [8]. Reduced CHS (crown height space) can have several negative effects, such as a reduced abutment height that could compromise long-term maintenance, insufficient bulk of restorative material for strength or aesthetics, and insufficient restoration retention. Abutments below 3 mm tall are best secured with screw retention for reliability. For abutments between 3 mm and 4 mm, you can opt for either screw retention or explore various cement types suitable for non-retrievable cementation. Abutments exceeding 4 mm in height can utilize retrievable cement for easy removal.

However, these procedures are complex, resource-intensive, and may necessitate sacrificing healthy dental tissues. Additionally, the invasive nature of wound healing and potential surgical complications further compound the challenges [9]. This case report presents one case study of the posterior region with less/limited interocclusal space using an endosteal implant with deep placement along with customization abutments with screw-retained for the final prosthesis.

## Case presentation

**Figure 1:**
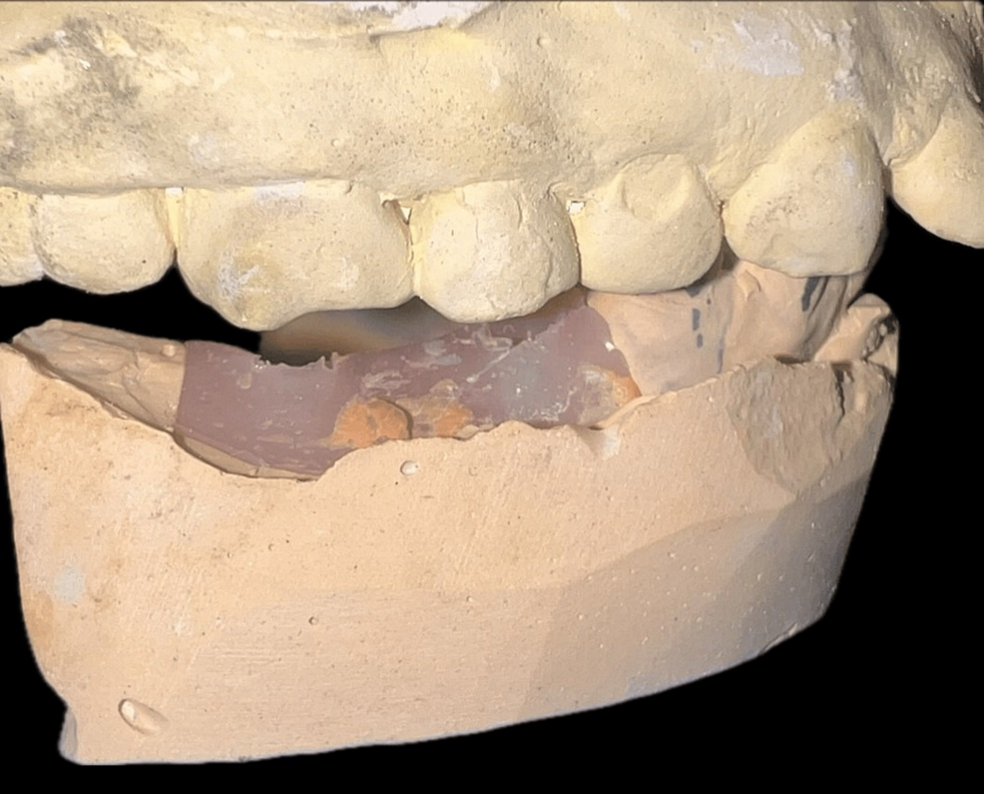
Limited interocclusal distance shown posteriorly between maxillary teeth and mandibular ridge

A 45-year-old female patient has visited the department of prosthodontics at Sharad Pawar Dental College and Hospital to replace missing 45, 46 and 47 (mandibular right 2nd premolar, 1st and 2nd molars). Intra-oral examination revealed interocclusal space near 45 and 46 is 3 mm and 47 is 2 mm, respectively (Figure 1).

**Figure 2:**
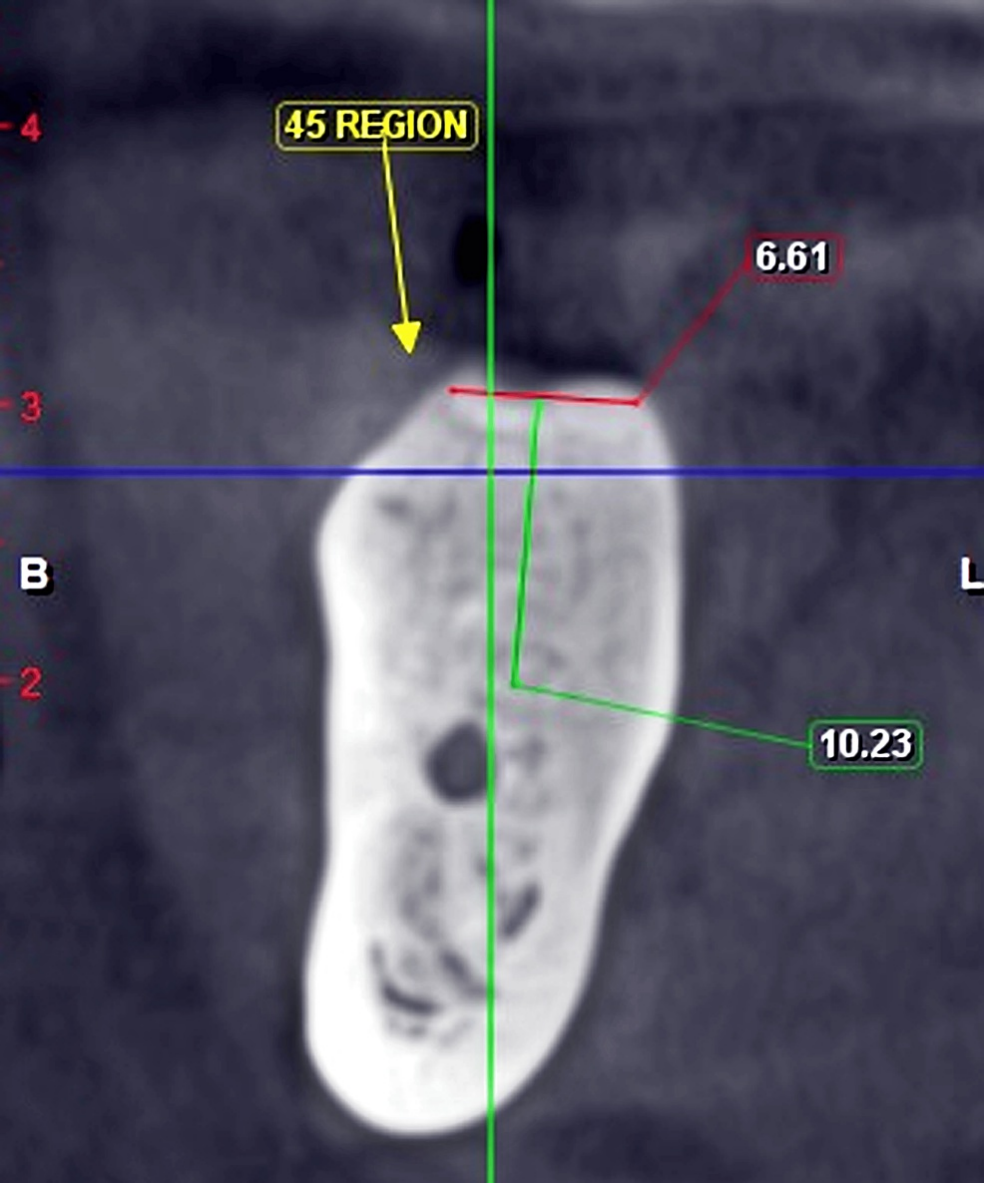
CBCT image a (sagittal section, 2 mm slice view) The cone beam computed tomography (CBCT) image shows available bone height and width in the 45 region.

**Figure 3:**
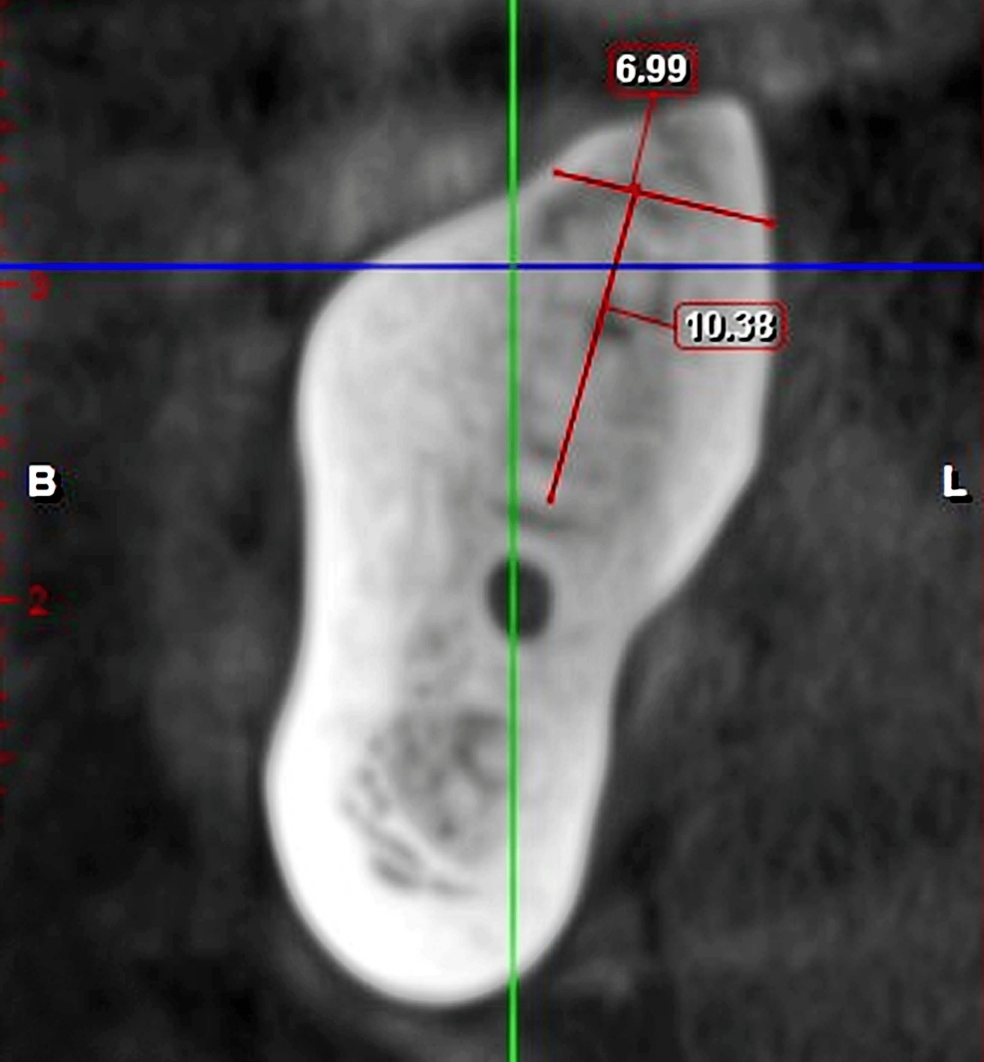
CBCT image 2 (sagittal section, 2 mm slice view) The cone beam computed tomography (CBCT) image shows available bone height and width in the 47 region.

Hence, there is an inadequate vertical dimension to replace the posterior teeth on the right mandibular region, as the ideal crown height should range from 4.23 to 3.67 for mandibular posteriors. On radiographic investigation, cone beam computed tomography (CBCT) showed adequate bone for endosteal implant placement (Figures 2-3). Consequently, after a thorough examination and considering the findings, the treatment plan was to place two endosteal implants and restore the lost interocclusal space. Restoring the lost vertical dimension while considering underlying conditions is crucial. A better treatment plan approach would be the deep placement of endosteal implants to regain the lost interocclusal space [10].

**Figure 4:**
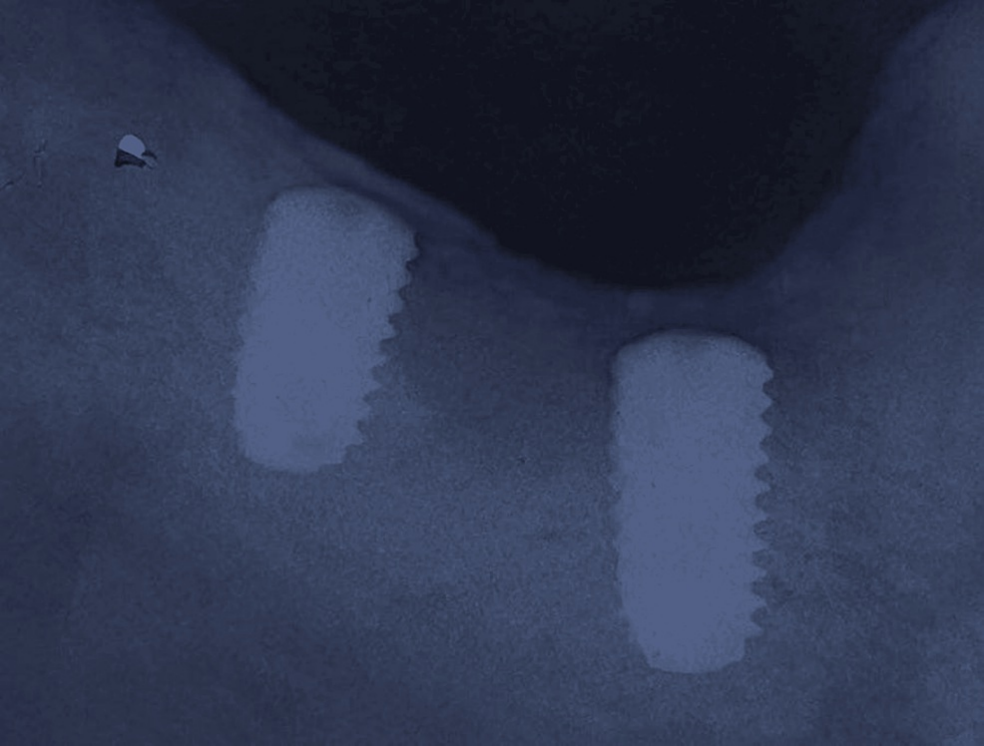
Three months post-surgical osseointegrated implants

The patient has been explained about the treatment plan and informed consent was obtained. Two deep endosteal implants (Osstem TS III implant system (Osstem Implant Co., Ltd., Seoul, Korea) dimensions are 45 region 5 mm width x 10 mm length, 47 region 5 mm width x 8.5 mm length) were inserted after the surgical procedure, which was carried out under local anesthesia using lignocaine and adrenaline at a ratio of 1:100,000. Post-surgery patients have received oral hygiene and post-surgical instructions along with standard antibiotic prophylaxis. For achieving osseointegration, the implants submerged were kept for three months before proceeding with the second stage of surgery to place healing abutments (Figure 4).

**Figure 5:**
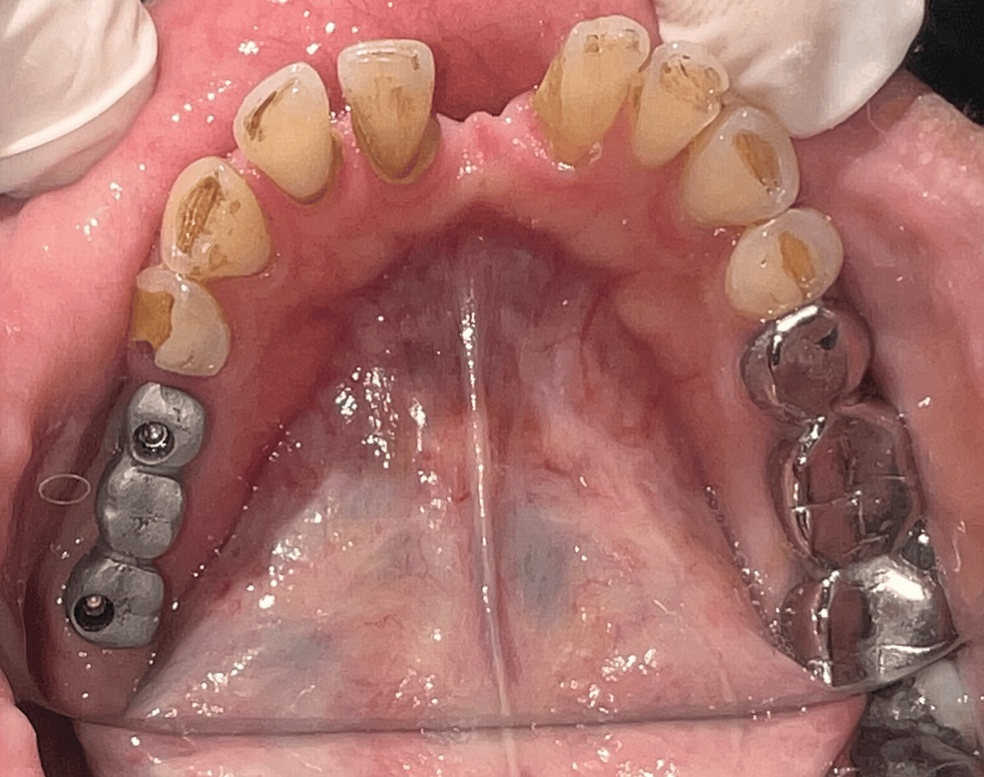
Screw-retained metal trail for posterior mandibular teeth

**Figure 6:**
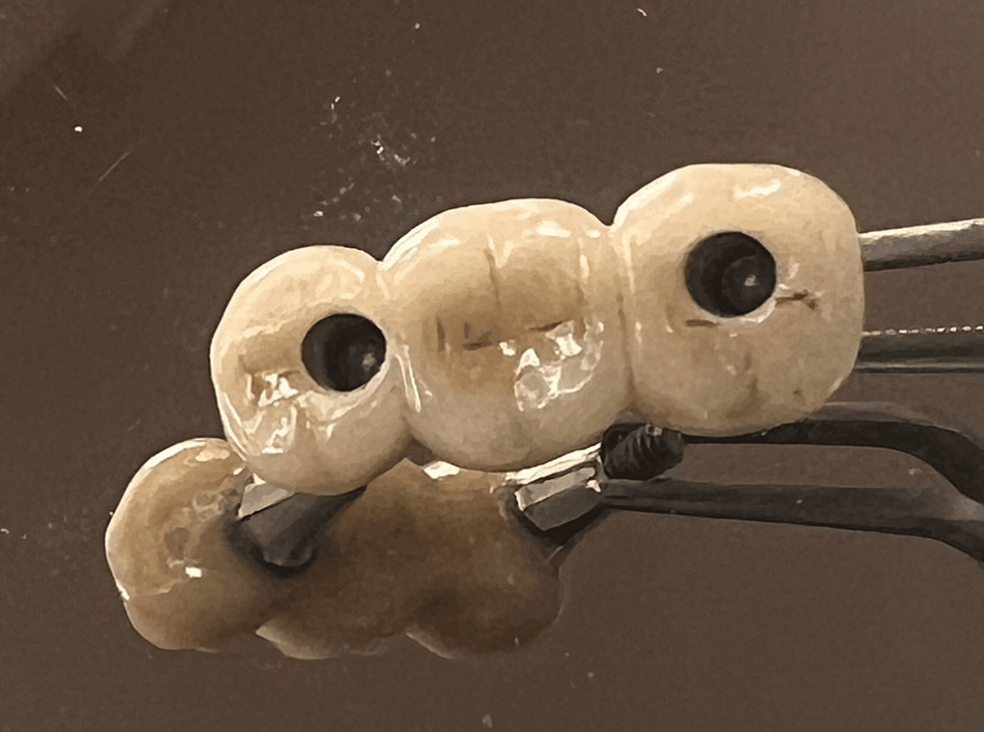
Screw retained implant fixed partial denture final prosthesis

At the 20-day follow-up, a closed tray impression was taken using Aquasil Ultra material (Dentsply Sirona, Pennsylvania, USA) and regular closed tray impression copings from the Osstem implant system. Due to the preplanned implant placement, the implants were positioned 1.5 mm below the bone crest level, and customized abutments were planned to ensure a minimum crown height of 4-5 mm for all posterior teeth. Intraoral verification of the customed screw-retained metal trail was performed to ensure proper fit and adequate space for ceramic layering (Figures 5-6).

**Figure 7:**
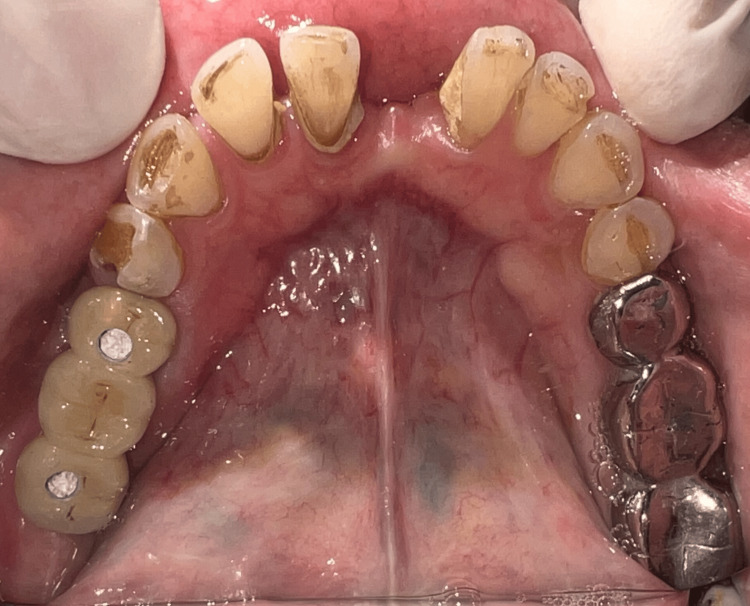
Post-insertion picture of screw-retained fixed partial denture

**Figure 8:**
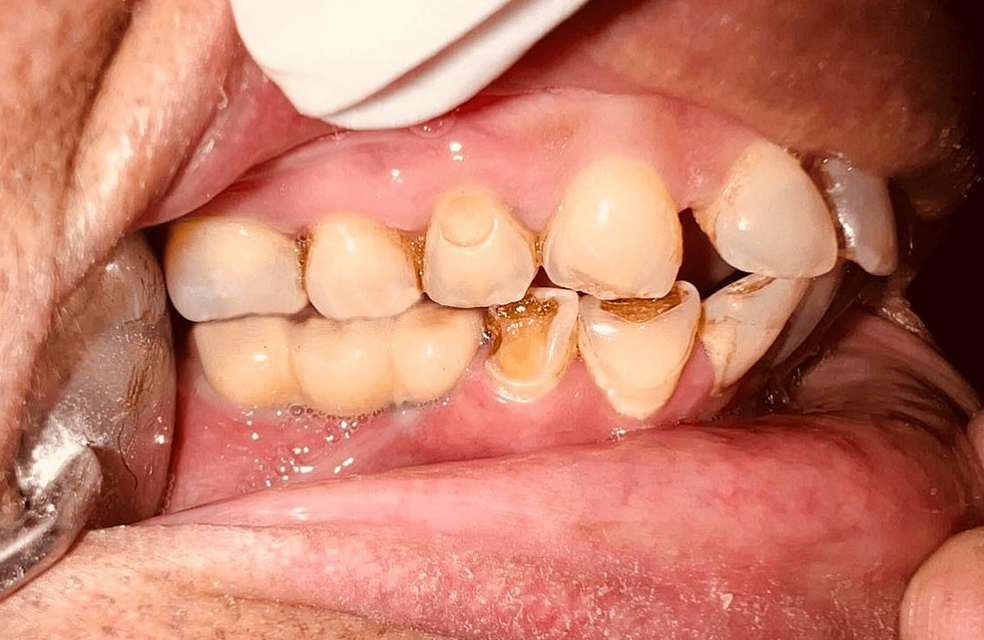
Facial view of post-insertion screw-retained fixed partial denture

A screw-retained fixed partial denture was inserted and verified for proper fit, and a torque of 30 N was applied, followed by packing the screw access holes with Teflon and restoring them with composite material (Figures 7-8). A post-insertion radiograph was taken to verify the placement and alignment of the abutments and prosthetic components with the implants.

Post-insertion oral hygiene maintenance instructions and regular yearly follow-up appointments for clinical and radiographic evaluation were provided. During these subsequent examinations, we conducted assessments on the marginal bone level surrounding the implants and evaluated factors such as mobility and other indicators of potential clinical issues to uphold the continued well-being and steadfastness of the implants [11].

## Discussion

The present case report outlines the management of decreased interocclusal space with deeply placed implants and implant-retained prostheses. Follow-up was conducted for one year and two months to assess clinical and radiographic outcomes. When dealing with decreased interocclusal distance in teeth in the posterior, the treatment plan can sometimes be unconventional. For example, opting for orthodontic tooth movement is often time-consuming and may not be feasible for many patients. On the other hand, crowns and more endodontic therapy may be required if the height of the opposing extruded teeth is lowered due to hypersensitivity. The last resort, surgical reconstruction via posterior maxillary segmental osteotomy, involves a more invasive procedure with potential complications, including postoperative infection of the surgical site, hemorrhage, vitality loss of the adjacent tooth, and bone fragment necrosis [12].

Any bone development on the implant platform can be carefully removed during the second stage of surgery by utilizing bone-cutting burs and a straight handpiece. This approach offers enhanced precision and minimizes invasiveness when compared to the traditional method of flattening the bone level before implant placement [13,14]. In the third stage, implementing an implant-retained fixed partial denture is strategically planned, given the constraints posed by the limited interocclusal distance. This choice presents several advantages, including the ease of prosthesis retrieval during routine hygiene maintenance, repair procedures, or necessary surgical interventions [15]. In instances where subgingival margins are present with cement-retained prostheses, during the cementation process, there is a risk of excess cement becoming compressed between the soft tissue and the implant [16,17]. As a result, this strategy aims to restore interocclusal space effectively. Specifically, subgingival margins are integrated into the treatment plan to elevate crown height and enhance retention, thereby contributing to a more successful treatment outcome.

## Conclusions

In conclusion, this case report demonstrates promising outcomes using the deeply placed Osstem TS III implant system. This endosteal implant, with its tapered design, provides excellent mechanical stability, making it suitable for patients with limited interocclusal space. Throughout the one-year and two-month follow-up duration, no indicators of failure were detected, underscoring the clinical success and functional efficacy of the locking-taper implants. These findings suggest that locking-taper implants may be viable for addressing such cases, particularly when used with screw-retained prostheses. However, further investigation with extended follow-up periods is imperative to corroborate these findings and ascertain the long-term viability of this approach. In essence, our study underscores the potential of locking-taper implants, particularly in conjunction with screw-retained prostheses, as a valuable solution in managing patients with restricted interocclusal space.

## References

[1] Najeeb S, Zafar MS, Khurshid Z, Siddiqui F (2016). Applications of polyetheretherketone (PEEK) in oral implantology and prosthodontics. J Prosthodont Res.

[2] Moraschini V, Poubel LA, Ferreira VF, Barboza Edos S (2015). Evaluation of survival and success rates of dental implants reported in longitudinal studies with a follow-up period of at least 10 years: a systematic review. Int J Oral Maxillofac Surg.

[3] Misch CE (2015). Dental implant prosthetics. https://shop.elsevier.com/books/dental-implant-prosthetics/misch/978-0-323-07845-0.

[4] Baeg S, On S, Lee J, Song S (2016). Posterior maxillary segmental osteotomy for management of insufficient intermaxillary vertical space and intermolar width discrepancy: a case report. Maxillofac Plast Reconstr Surg.

[5] Lee HE, Lee KT, Tseng YC, Huang IY, Chen CM (2008). Interdisciplinary management of unfavorable posterior intermaxillary space. Br J Oral Maxillofac Surg.

[6] Geckili O, Sakar O, Yurdakuloglu T, Firatli S, Bilhan H, Katiboglu B (2011). Multidisciplinary management of limited interocclusal space: a clinical report. J Prosthodont.

[7] Chun YS, Row J, Yang SJ, Cha HS, Han JS (2000). Management of extruded maxillary molars to accommodate a mandibular restoration: a clinical report. J Prosthet Dent.

[8] Chen DL, Xu JM, Wu ZQ, Wang QN, Wang P, Tang CB (2017). A clinical analysis of screw-retained implant-supported casted integrated abutment crowns in the molar region with limited interocclusal space. Shanghai Kou Qiang Yi Xue.

[9] Lombardo G, Corrocher G, Pighi J, Faccioni F, Rovera A, Marincola M, Nocini PF (2014). The impact of subcrestal placement on short locking-taper implants placed in posterior maxilla and mandible: a retrospective evaluation on hard and soft tissues stability after 2 years of loading. Minerva Stomatol.

[10] Moreno-Hay I, Okeson JP (2015). Does altering the occlusal vertical dimension produce temporomandibular disorders? A literature review. J Oral Rehabil.

[11] Linkevicius T, Vindasiute E, Puisys A, Peciuliene V (2011). The influence of margin location on the amount of undetected cement excess after delivery of cement-retained implant restorations. Clin Oral Implants Res.

[12] Cioffi I, Farella M, Festa P, Martina R, Palla S, Michelotti A (2015). Short-term sensorimotor effects of experimental occlusal interferences on the wake-time masseter muscle activity of females with masticatory muscle pain. J Oral Facial Pain Headache.

[13] Huang B, Meng H, Piao M, Xu L, Zhang L, Zhu W (2012). Influence of placement depth on bone remodeling around tapered internal connection implant: a clinical and radiographic study in dogs. J Periodontol.

[14] Lee EH, Ryu SM, Kim JY, Cho BO, Lee YC, Park YJ, Kim SG (2010). Effects of installation depth on survival of an hydroxyapatite-coated Bicon implant for single-tooth restoration. J Oral Maxillofac Surg.

[15] Albrektsson T, Zarb GA (1998). Determinants of correct clinical reporting. Int J Prosthodont.

[16] Wilson TG Jr (2009). The positive relationship between excess cement and peri-implant disease: a prospective clinical endoscopic study. J Periodontol.

[17] Frisch E, Ratka-Krüger P, Weigl P, Woelber J (2016). Extraoral cementation technique to minimize cement-associated peri-implant marginal bone loss: can a thin layer of zinc oxide cement provide sufficient retention?. Int J Prosthodont.

